# Supplementary material for: Clinical and genomic features of Chinese lung cancer patients with germline mutations
Source: Nat Commun. 2022 Mar 10;13:1268. doi: 10.1038/s41467-022-28840-5 (PMC8913621; doi:10.1038/s41467-022-28840-5)
Supplement: Supplementary file 1 — Supplementary Information [file 41467_2022_28840_MOESM1_ESM.pdf]

Supplementary information

Clinical and genomic features of Chinese lung cancer patients with germline mutations

Peng et al.

Supplementary Figure

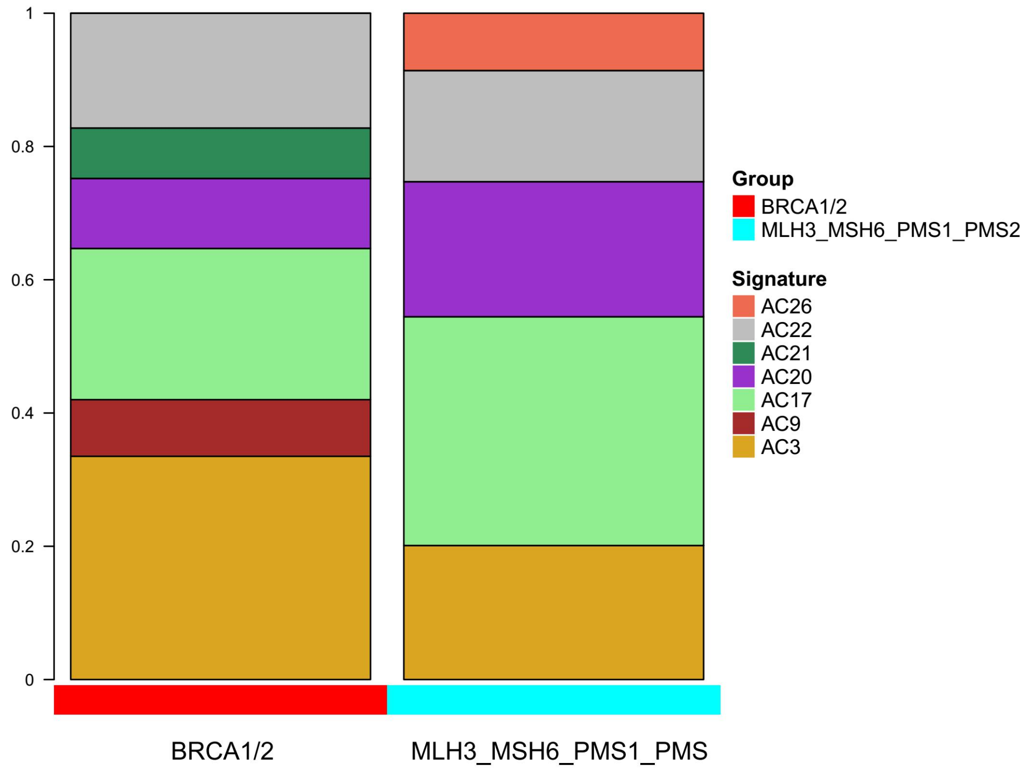

Supplementary Figure 1 COSMIC mutational signatures in samples from patients with *BRCA1*/2 germline mutations versus mismatch repair (MMR) germline mutations. The stacked bar plot represents fraction of mutations associated with each signature. The mutational signature.

Supplementary Tables

Supplementary Table 1. Tumor samples with LOH of genes with germline mutations

LOH region in tumor						Germline mutation			
ID	chrom	start	end	tcn.em	lcn.em	Gene symbol	start	end	Mutation
P016	17	33428100	37853048	1	0	***RAD51D***	33434458	33434458	c.270_271dupTA (p.K91Ifs*13)
P021	13	19751500	111371892	1	0	***BRCA2***	32910550	32910555	c.2059_2063delGATTA (p.D687*fs*1)
P023	11	104799600	108296300	1	0	***ATM***	108151841	108151842	c.3523delG (p.A1175Pfs*6)
P029	13	19751544	49051700	1	0	***BRCA2***	32954049	32954050	c.9117G>A (p.(P3039=))
P087	13	32889400	49047700	1	0	***BRCA2***	32954179	32954180	c.9154C>T (p.R3052W)
P089	13	19751544	111371892	1	0	***BRCA2***	32911708	32911709	c.3217C>T (p.Q1073*)
P093	13	19751544	52059000	1	0	***BRCA2***	32914640	32914640	c.6149dupT (p.N2051Kfs*9)
P096	17	1761200	80790000	2	0	***TP53***	7578411	7578412	c.518T>C (p.V173A)

LOH, loss of heterozygosity. Chrom, Chromosome. Tcn.em, estimated total copy number. Lcn.em, estimated minor copy number.

Supplementary Table 2. Tumor sample with lost of function mutations in genes with germline mutation.

ID	Sample	Germline	Somatic
P025	FFPE	NM_000546.5(TP53): c.541C>T (p.R181C)	TP53, c.574C>T p.Q192*
P010	FFPE	NM_000548.3(TSC2): c.2356-2A>C	TSC2, c.889T[6>10] p.V299Ffs*40
P066	FFPE	NM_000059.3(BRCA2): c.8400_8402delTTTinsAAAA (p.F2801Kfs*11)	BRCA2, c.316+1G>A

FFPE, formalin fixed paraffin-embedded sample.

Supplementary Table 3. 75 genes analysed for germline mutations both in Chinese cohort and TCGA Cohort

Germline genes shared in this study and TCGA study
BRCA2
FANCA
ATM
MUTYH
RAD51D
BLM
TP53
FANCD2
BRIP1
BRCA1
MSH6
CHEK2
PMS2
PMS1
PALB2
RAD51C
SDHA
NBN
TSC2
FANCC
BAP1
CDH1
FLCN
NF1
RUNX1
RET
ERCC3
FANCG
VHL
HNF1A
ALK
APC
AXIN2
BMPR1A
CBL
CDC73
CDK4
CDKN2A
DICER1
EGFR

FH
HRAS
KIT
MAX
MEN1
MET
NF2
PDGFRA
PRKAR1A
PTCH1
PTEN
PTPN11
RB1
SDHAF2
SDHB
SDHC
SDHD
SMAD4
SMARCA4
SMARCB1
STK11
SUFU
TSC1
WT1
FANCM
MITF
PAX5
EPCAM
ATR
CDKN1B
MLH1
MSH2
POLD1
POLE
TMEM127

Supplementary Table 4. Comparison of germline mutation prevalence between Chinese lung cancer and TCGA lung cancer cohorts.

	All patients			LUAD			LUSC		
	Chinese	TCGA	*p*	Chinese	TCGA	*p*	Chinese	TCGA	*p*
Total number	1794	1017		1223	518		170	499	
P/LP+ patients (n/%)	106 (5.91)	64 (6.29)		75 (6.13)	37 (7.14)		10 (5.88)	27 (5.4)	
P/LP- patients	1688	953	0.743	1148	481	0.497	160	472	0.970
	within 75 predisposition genes covered in both cohorts								
P/LP+ patients (n,%)	94 (5.24)	34 (3.34)		66 (5.67)	17 (3.28)		9 (5.63)	17 (3.4)	
P/LP patients	1700	983	0.026	1157	501	0.077	161	482	0.384

P-value is calculated with Chi-square test.

Supplementary Table 5. Number of Patients with germline mutations in certain genes in Chinese cohort and TCGA.

Germline mutation	All patients included			LUAD			LUSC		
	Chinese N=1794	TCGA N=1017	***p***	Chinese N=1223	TCGA N=518	***p***	Chinese N=170	TCGA N=499	***p***
ATM									
yes	7	2	0.502	3	2	0.635	2	**0**	0.064
no	1787	1015		1220	516		168	499	
BAP1									
yes	1	**0**	1	1	**0**	1	**0**	**0**	
no	1793	1017		1222	518		170	499	
BLM									
yes	5	2	1	3	1	1	2	1	0.161
no	1789	1015		1220	517		168	498	
BRCA1									
yes	4	6	0.183	4	2	1	**0**	4	0.577
no	1790	1011		1219	516		170	495	
BRCA2									
yes	14	1	**0.015**	11	0	**0.041**	**0**	1	1
no	1780	1016		1212	518		170	498	
BRIP1									
yes	5	**0**	0.166	3	**0**	0.559	**0**	**0**	
no	1789	1017		1220	518		170	499	
CDH1									
yes	1	**0**	1	**0**	**0**		**0**	**0**	
no	1793	1017		1223	518		170	499	
CHEK2									
yes	3	3	0.674	3	3	0.37	**0**	**0**	
no	1791	1014		1220	515		170	499	
ERCC3									
yes	**0**	2	0.131	**0**	1	0.298	**0**	1	1
no	1794	1015		1223	517		170	498	
FANCA									
yes	9	5	1	7	0	0.112	1	5	1
no	1785	1012		1216	518		169	494	
FANCC									
yes	1	1	1	1	0	1	**0**	1	1
no	1793	1016		1222	518		170	498	
FANCD2									
yes	5	**0**	0.166	4	**0**	0.325	**0**	**0**	
no	1789	1017		1219	518		170	499	
FANCG									

yes	**0**	2	0.131	**0**	1	0.298	**0**	1	1
no	1794	1015		1223	517		170	498	
FLCN									
yes	1	**0**	1	**0**	**0**		**0**	**0**	
no	1793	1017		1223	518		170	499	
MSH6									
yes	4	**0**	0.304	1	**0**	1	2	**0**	0.064
no	1790	1017		1222	518		168	499	
MUTYH									
yes	6	1	0.433	4	1	1	**0**	**0**	
no	1788	1016		1219	517		170	499	
NBN									
yes	2	2	0.624	**0**	2	0.088	**0**	**0**	
no	1792	1015		1223	516		170	499	
NF1									
yes	1	**0**	1	**0**	**0**		1	**0**	0.254
no	1793	1017		1223	518		169	499	
PALB2									
yes	3	**0**	0.558	3	**0**	0.559	**0**	**0**	
no	1791	1017		1220	518		170	499	
PMS1									
yes	3	**0**	0.558	3	**0**	0.559	**0**	**0**	
no	1791	1017		1220	518		170	499	
PMS2									
yes	3	1	1	3	0	0.559	**0**	1	1
no	1791	1016		1220	518		170	498	
RAD51C									
yes	3	**0**	0.558	2	**0**	1	**0**	**0**	
no	1791	1017		1221	518		170	499	
RAD51D									
yes	7	**0**	0.054	4	**0**	0.325	1	**0**	0.254
no	1787	1017		1219	518		169	499	
RET									
yes	**0**	3	**0.047**	**0**	2	0.088	**0**	1	1
no	1794	1014		1223	516		170	498	
RUNX1									
yes	1	**0**	1	1	**0**	1	**0**	**0**	
no	1793	1017		1222	518		170	499	
SDHA									
yes	3	**0**	0.558	3	**0**	0.559	**0**	**0**	
no	1791	1017		1220	518		170	499	

TP53									
yes	5	2	1	4	1	1	**0**	1	1
no	1789	1015		1219	517		170	498	
TSC2									
yes	2	**0**	0.538	2	**0**	1	**0**	**0**	
no	1792	1017		1221	518		170	499	
VHL									
yes	**0**	1	0.362	**0**	1	0.298	**0**	**0**	
no	1794	1016		1223	517		170	499	

P - value is calculated with Fisher's exact test.

Supplementary Table 6. Correlation between patient clinical information and germline mutation prevalence.

	Univariate analysis		Multivariate analysis	
	**OR（95%CI）**	***p*-value**	**OR（95%CI）**	***p*-value**
		Germline mutation		
Gender (Male vs. Female)	1.123(0.754-1.673)	0.567	1.270(0.803-2.007)	0.307
Histology (LUAD vs. LUSC)	0.964(0.488-1.906)	0.917	1.249(0.612-2.550)	0.541
Age of diagnosis	0.987(0.970-1.004)	0.124	0.995(0.975-1.015)	0.622
Stage (IV vs I-III)	1.450(0.776-2.709)	0.244	1.531(0.813-2.884)	0.298

Odds ratios, two sided *p*-value and 95% confidence intervals were calculated using logistic regression models. OR, odds ratio. All factors were included in both univariate analysis and multivariate logistic regression models. LUAD, lung adenocarcinoma. LUSC, lung squamous carcinoma.

Supplementary Table 7. *MET* somatic mutations

ID	Germline mutation	Somatic MET mutation or CNV	Function
C0042	negative	4.123873275	CNA
C0062	negative	c.100G>C	missense
C0116	negative	2.303	CNA
C0332	negative	c.4087G>A	missense
C0381	negative	1.667	CNA
C0431	negative	4.28709385	CNA
C0462	negative	c.3028+1G>A	14 exon skkiping
C0478	negative	c.500_513delAAGAGCCCAGCCAG	frameshift
C0478	negative	c.2888-24_2888-13delCTCTTTCTTTCT	14 exon skkiping
C0508	negative	3.349	CNA
C0509	negative	c.3822G>T	missense
C0509	negative	c.1660_1661delGGinsTT	missense
C0510	negative	c.3028+2T>G	14 exon skkiping
C0548	negative	c.3028+1_3028+3delGTAinsAT	14 exon skkiping
C0564	negative	4.383	CNA
C0572	negative	c.3028G>C	missense
C0572	negative	4.109605758	CNA
C0589	negative	c.3028+1G>T	14 exon skkiping
C0639	negative	c.2594T>C	missense
C0647	negative	4.349953563	CNA
C0671	negative	c.3028+3A>G	14 exon skkiping
C0744	negative	c.3028+2T>A	14 exon skkiping
C0808	negative	c.2888-17_2888-7delTTTCTCTCTGT	14 exon skkiping
C0932	negative	c.423C>A	nonsense
C0937	negative	c.2294A>G	missense
C0948	negative	c.3028+1G>A	14 exon skkiping
C0952	negative	c.3028+1G>A	14 exon skkiping
C0952	negative	c.3980G>T	missense
C0963	negative	2.35	CNA
C0976	negative	3.904	CNA
C1002	negative	c.2413T>A	missense
C1074	negative	10.951	CNA
C1078	negative	c.3504C>A	missense
C1210	negative	c.2971C>T	missense
C1216	negative	c.1623_1627delCCACG	frameshift
C1238	negative	c.3028+1G>A	14 exon skkiping
C1263	negative	c.385C>G	missense
C1273	negative	c.3280C>T	missense
C1289	negative	c.3028G>C	missense
C1339	negative	c.2960G>T	missense

C1339	negative	c.1448C>G	missense
C1405	negative	c.845G>T	missense
C1418	negative	1.719	CNA
C1529	negative	c.3865A>G	missense
C1554	negative	c.3028G>C	missense
C1571	negative	c.731C>G	missense
C1599	negative	c.1140A>C	missense
P0009	positive	c.1595C>T	missense
P0029	positive	c.3028+4delGGTATinsA	14 exon skkiping
P0075	positive	6.973388305	CNA
P0077	positive	c.611C>T	missense
P0089	positive	c.2888-19_2888delTCTTTCTCTCTGTTTTAAGA	14 exon skkiping
P0095	positive	c.3028+3A>T	14 exon skkiping
P0102	positive	c.2888-15_2888-1delTCTCTCTGTTTTAAG	14 exon skkiping
P0102	positive	c.2913C>A	nonsense

Supplementary Table 8. Somatic mutation prevalence in patients with germline mutation

Somatic mutation	Germline P/LP mutation + n=92 (n,%)	Germline P/LP mutation - n=1434 (n,%)	*p*-value
TP53			0.017*
mutant	42 (45.7)	845 (58.9)	
wildtype	50	589	
EGFR			0.720*
mutant	41 (44.6)	675 (47.1)	
wildtype	51	759	
KRAS			0.015*
mutant	15 (16.3)	119 (8.3)	
wildtype	77	1315	
c-MET			0.027#
mutant	7 (7.6)	43 (3.0)	
wildtype	86	1391	
c-MET CNV+	1 (1.1)	12 (0.84)	0.556#
c-MET CNV -	91	1422	
c-MET Skipping+	4 (4.35)	11 (0.77)	0.010#
c-MET Skipping-	88	1423	
ALK			1#
mutant	5 (5.4)	83 (5.8)	
wildtype	87	1351	
ROS-1			0.600#
mutant	5 (5.4)	63 (4.4)	
wildtype	87	1371	
ERBB2			1#
mutant	4 (4.3)	73 (5.1)	
wildtype	88	1361	
RET			0.255#
mutant	1 (1.1)	57 (4.0)	
wildtype	91	1377	
BRAF			0.766#
mutant	2 (2.2)	49 (3.4)	
wildtype	90	1383	

Somatic gene mutations included SNV/indel, CNV and SV.

* *p*-value was calculated with Chi-square test.

p-value was calculated with Fisher's exact test

Supplementary Table 9. The comparison of clinical features in TCGA cohort with Chinese cohort.

	TCGA	Chinese	*p*-value
Sample size, *N*(%)			
All	1017	1794	
LUAD	518 (51)	1223(68.2)	
LUSC	499 (49)	170(9.5)	
SCLC	0	44(2.5)	
Other histology	0	44(2.5)	
NSCLC-NOS	0	313(17.4)	
Age, years(mean±SD)			
All	66.3±9.4	60.0±11.5	1.42E-48
LUAD	65.3±10.0	58.5±11.1	5.86E-32
LUSC	67.3±8.6	63.2±11.1	7.01E-07
SCLC	NA	60.2±10.5	
Other histology	NA	55.3±11.7	
NSCLC-NOS	NA	63.6±12.6	
Female proportion(%)			
All	40.0(411/1027)	42.8(767/1792)	0.161
LUAD	53.6 (280/522)	47.8(584/1223)	0.028
LUSC	26.0 (131/504)	13.5 (23/170)	0.001
SCLC	0	25 (11/44)	
Other histology	0	38.6 (17/44)	
NSCLC-NOS	0	42.4(132/311)	2 NA

Other histology type included: large cell neuroendocrine carcinoma, adenosquamous carcinoma, sarcomatoid carcinoma, pleomorphic carcinoma, poorly differentiated carcinoma, mucoepidermoid carcinoma, lymphoepithelioid carcinoma, etc. Age difference between TCGA cohort and Chinese cohort was calculated with Mann-Whitney test. Female proportion difference was calculated with Chi-square test. NA, not available.

Supplementary Table 10. 94 cancer susceptibility genes of germline

Gene list
AKT1
ALK
APC
ATM
ATR
AXIN2
BAP1
BARD1
BLM
BMPR1A
BRCA1
BRCA2
BRIP1
CBL
CDC73
CDH1
CDK4
CDKN1B
CDKN2A
CHEK1
CHEK2
DICER1
EGFR
EPCAM
ERCC3
FAM175A
FANCA
FANCC
FANCD2
FANCG
FANCM
FGFR3
FH
FLCN
GALNT12
HNF1A
HOXB13
HRAS
KIT
MAX

MEN1
MET
MITF
MLH1
MLH3
MRE11A
MSH2
MSH3
MSH6
MUTYH
NBN
NF1
NF2
NSD1
NTRK1
PALB2
PAX5
PDGFRA
PIK3CA
PMS1
PMS2
POLD1
POLE
PRKAR1A
PTCH1
PTCH2
PTEN
PTPN11
RAD50
RAD51
RAD51B
RAD51C
RAD51D
RB1
RET
RUNX1
SDHA
SDHAF2
SDHB
SDHC
SDHD
SLX4
SMAD4

SMARCA4
SMARCB1
STK11
SUFU
TMEM127
TP53
TSC1
TSC2
VHL
WT1
XRCC2
