# Supplementary material for: Clinical and genomic features of Chinese lung cancer patients with germline mutations
Source: Nat Commun. 2022 Mar 10;13:1268. doi: 10.1038/s41467-022-28840-5 (PMC8913621; doi:10.1038/s41467-022-28840-5)
Supplement: Supplementary file 3 — Description of Additional Supplementary Files [file 41467_2022_28840_MOESM3_ESM.pdf]

Description of Additional Supplementary Files

File Name: Supplementary Data 1
Description: Annotation of 111 P/LP germline mutations in 106 lung cancers.

File Name: Supplementary Data 2
Description: Clinical information and germline mutations of 1794 lung cancer patients.

File Name: Supplementary Data 3
Description: 1021 genes in the NGS panel.

File Name: Supplementary Data 4
Description: Functional somatic SNV and indel.

File Name: Supplementary Data 5
Description: Somatic copy number variants (CNV).

File Name: Supplementary Data 6
Description: Somatic structural variants (SV).
