# Supplementary material for: Clinical and genomic features of Chinese lung cancer patients with germline mutations
Source: Nat Commun. 2022 Mar 10;13:1268. doi: 10.1038/s41467-022-28840-5 (PMC8913621; doi:10.1038/s41467-022-28840-5)
Supplement: Supplementary file 10 — Reporting summary [file 41467_2022_28840_MOESM10_ESM.pdf]

nature research

Corresponding author(s): Lin Wu, Jianjun Zhang, Fenglei Yu

Last updated by author(s): Sep 7, 2020

Reporting Summary

Nature Research wishes to improve the reproducibility of the work that we publish. This form provides structure for consistency and transparency in reporting. For further information on Nature Research policies, see our Editorial Policies and the Editorial Policy Checklist.

Statistics

For all statistical analyses, confirm that the following items are present in the figure legend, table legend, main text, or Methods section.

n/a	Confirmed	
☐	☒	The exact sample size (*n*) for each experimental group/condition, given as a discrete number and unit of measurement
☐	☒	A statement on whether measurements were taken from distinct samples or whether the same sample was measured repeatedly
☐	☒	The statistical test(s) used AND whether they are one- or two-sided<br>*Only common tests should be described solely by name; describe more complex techniques in the Methods section.*
☐	☒	A description of all covariates tested
☐	☒	A description of any assumptions or corrections, such as tests of normality and adjustment for multiple comparisons
☐	☒	A full description of the statistical parameters including central tendency (e.g. means) or other basic estimates (e.g. regression coefficient) AND variation (e.g. standard deviation) or associated estimates of uncertainty (e.g. confidence intervals)
☐	☒	For null hypothesis testing, the test statistic (e.g. *F*, *t*, *r*) with confidence intervals, effect sizes, degrees of freedom and *P* value noted<br>*Give P values as exact values whenever suitable.*
☒	☐	For Bayesian analysis, information on the choice of priors and Markov chain Monte Carlo settings
☒	☐	For hierarchical and complex designs, identification of the appropriate level for tests and full reporting of outcomes
☒	☐	Estimates of effect sizes (e.g. Cohen's *d*, Pearson's *r*), indicating how they were calculated

Our web collection on statistics for biologists contains articles on many of the points above.

Software and code

Policy information about availability of computer code

Data collection	This study does not use any software to collect open source data.
Data analysis	Base-calling was conducted through the Illumina analysis pipeline (CASAVA Version 1.8). GATK (Version 3.6) was used to detect single-nucleotide variants (SNVs) and small insertions and deletions (indels) from germline DNA. Somatic SNVs in tumor DNA were called using MuTect (Version 1.4) . GATK(Version 3.6) was used to identify indels. Somatic copy-number variation (CNV) were identified with CONTRA (Version 2.0.8), Loss of heterozygosity (LOH) was analyzed with Facets (Version 1.0.1). The mean pairwise Fst differences between different populations were calculated using EIGENSOFT (Version 7.2.1). Principal component analysis (PCA) was performed using PLINK (Version1.9) and EIGENSOFT (Version 7.2.1). Signature analysis was performed with R package "YAPSA". All statistical analysis was performed with SPSS (v.23.0; STATA, College Station, TX, USA) or GraphPad Prism (v. 6.0; GraphPad Software, La Jolla, CA, USA) software.

For manuscripts utilizing custom algorithms or software that are central to the research but not yet described in published literature, software must be made available to editors and reviewers. We strongly encourage code deposition in a community repository (e.g. GitHub). See the Nature Research guidelines for submitting code & software for further information.

Data

Policy information about availability of data

All manuscripts must include a data availability statement. This statement should provide the following information, where applicable:

- Accession codes, unique identifiers, or web links for publicly available datasets
- A list of figures that have associated raw data
- A description of any restrictions on data availability

Patient deidentified clinical and mutation data (both germline and somatic mutations) were provided in the Supplementary data 1-6. The VCF data that support the findings of this study have been deposited in China National Gene Bank database (https://db.cngb.org/cnsa/) with accession number CNP0001060.

The reference genome used in this study was GRCh37/hg19. Public available datasets used in this study are listed below:
1000 Genomes Project (http://browser.1000genomes.org),
Clingen data base (https://www.clinicalgenome.org/),
ClinVar database(http://www.ncbi.nlm.nih.gov/clinvar),
COSMIC (https://cancer.sanger.ac.uk/cosmic),
dbSNP (http://www.ncbi.nlm.nih.gov/snp),
Exome Aggregation Consortium (http://exac.broadinstitute.org/),
Genome Aggregation Database (GnomAD) (http://gnomad.broadinstitute.org/),
Human Gene Mutation Database (HGMD) (http://www.hgmd.org),
NHLBI GO Exome Sequencing Project (ESP), (http://evs.gs.washington.edu/EVS/),
The Cancer Genome Atlas (TCGA), (https://portal.gdc.cancer.gov/),
The summary information from The China Metabolic Analytics Project (ChinaMAP), including the position, reference allele, mutated allele and allele frequencies of variants could be accessed through the ChinaMAP browser (www.mBiobank.com).
Researchers can gain access to the data online.

Field-specific reporting

Please select the one below that is the best fit for your research. If you are not sure, read the appropriate sections before making your selection.

☒ Life sciences ☐ Behavioural & social sciences ☐ Ecological, evolutionary & environmental sciences

For a reference copy of the document with all sections, see nature.com/documents/nr-reporting-summary-flat.pdf

Life sciences study design

All studies must disclose on these points even when the disclosure is negative.

Sample size	Sample size was not determined before the study. Cohort in this retrospective study included 1,794 lung cancer patients, who were subjected to target capture next generation sequencing (NGS) between December 2017 and August 2018 from three hospitals as part of the clinical care. TCGA lung cancer cohort included a thousand patients, sample size of this study (n=1794) was comparable for describe a germline mutation landscape between races.
Data exclusions	Patients diagnosed with lung cancer and paired normal-tumor sample sequenced were included. The germline mutations data from all 1794 patients were collected for analysis. As small cell lung cancer shows different somatic mutational landscape with non-small cell lung cancer, and too small sample size of SCLC (n=44) were included, only somatic data of NSCLCs were futher analysed. For somatic mutation analysis, to avoid false negative results ascribed to low tumor content in the specimens, 224 patients (including 187 with liquid biopsy samples and 37 with FFPE samples) without any somatic mutation detected were excluded for somatic mutation landscape analysis.
Replication	Since our assay is a clinical assay that have been extensively utilized and validated during R&D and subsequent clinical use, we initially did not perform independent validation for these data. However, we made extensive effort to obtain DNA to validate the data used in this study and were able to find two specimens (P001 positive for BRCA1 c.1961dupA (p.Y655Vfs*18) and P020 for BRCA2 c.6031T[2] (p.S2012Qfs*5)) for Sanger sequencing.<br>For somatic mutation landscape difference between patients with P/LP germline mutation and those without, multivariate analysis adjusting for gender, age and histology etc. were performed to verify the results.
Randomization	NA. All eligible patients/samples were included.
Blinding	This is a retrospective study, there are no instances of grouping or blingding based on the choice of the investigators.

Reporting for specific materials, systems and methods

We require information from authors about some types of materials, experimental systems and methods used in many studies. Here, indicate whether each material, system or method listed is relevant to your study. If you are not sure if a list item applies to your research, read the appropriate section before selecting a response.

Materials & experimental systems

n/a	Involved in the study	
☒	☐	Antibodies
☒	☐	Eukaryotic cell lines
☒	☐	Palaeontology and archaeology
☒	☐	Animals and other organisms
☐	☒	Human research participants
☒	☐	Clinical data
☒	☐	Dual use research of concern

Methods

n/a	Involved in the study	
☒	☐	ChIP-seq
☒	☐	Flow cytometry
☒	☐	MRI-based neuroimaging

Human research participants

Policy information about studies involving human research participants

Population characteristics	All patients were from China, with median age of 60 (range 16-94) and the ratio of male to female is 1025:767 (NA=2). All patients were diagnosed with lung cancer, and further treated as precision medicine according to guideline in real-world setting.
Recruitment	No patients were recruited particularly for this study. Patients were treated as standard of care and biopsy specimens were analyzed. One caveat is that compared to TCGA cohorts, patients in the current Chinese cohort were younger, with more female patients and stage IV diseases (Supplementary Table 9). These important differences could have potentially confounded the observed higher incidence of P/LP germline mutations in the Chinese cohort. However, in the current cohort, incidence of P/LP mutations did not seem to correlate with gender, age, histology or stage.
Ethics oversight	The study was approved by the Ethics Committee of Hunan Cancer Hospital and all participants signed a written informed consent.

Note that full information on the approval of the study protocol must also be provided in the manuscript.
